# Efficacy of Δ^9^ -Tetrahydrocannabinol (THC) Alone or in Combination With a 1:1 Ratio of Cannabidiol (CBD) in Reversing the Spatial Learning Deficits in Old Mice

**DOI:** 10.3389/fnagi.2021.718850

**Published:** 2021-08-30

**Authors:** Prakash Nidadavolu, Andras Bilkei-Gorzo, Michael Krämer, Britta Schürmann, Michela Palmisano, Eva C. Beins, Burkhard Madea, Andreas Zimmer

**Affiliations:** ^1^Institute of Molecular Psychiatry, Medical Faculty, University of Bonn, Bonn, Germany; ^2^Institute of Forensic Medicine, Medical Faculty, University of Bonn, Bonn, Germany; ^3^Institute of Human Genetics, Medical Faculty, University of Bonn, Bonn, Germany

**Keywords:** cognition, aging, Δ^9^-tetrahydrocannabinol, cannabidiol, endocannabinoid system

## Abstract

Decline in cognitive performance, an aspect of the normal aging process, is influenced by the endocannabinoid system (ECS). Cannabinoid receptor 1 (CB1) signaling diminishes with advancing age in specific brain regions that regulate learning and memory and abolishing CB1 receptor signaling accelerates cognitive aging in mice. We recently demonstrated that prolonged exposure to low dose (3 mg/kg/day) Δ^9^-tetrahydrocannabinol (THC) improved the cognitive performances in old mice on par with young untreated mice. Here we investigated the potential influence of cannabidiol (CBD) on this THC effect, because preclinical and clinical studies indicate that the combination of THC and CBD often exhibits an enhanced therapeutic effect compared to THC alone. We first tested the effectiveness of a lower dose (1 mg/kg/day) THC, and then the efficacy of the combination of THC and CBD in 1:1 ratio, same as in the clinically approved medicine Sativex^®^. Our findings reveal that a 1 mg/kg/day THC dose still effectively improved spatial learning in aged mice. However, a 1:1 combination of THC and CBD failed to do so. The presence of CBD induced temporal changes in THC metabolism ensuing in a transient elevation of blood THC levels. However, as CBD metabolizes, the inhibitory effect on THC metabolism was alleviated, causing a rapid clearance of THC. Thus, the beneficial effects of THC seemed to wane off more swiftly in the presence of CBD, due to these metabolic effects. The findings indicate that THC-treatment alone is more efficient to improve spatial learning in aged mice than the 1:1 combination of THC and CBD.

## Introduction

Although discreetly, the physiological aging process is associated with a decline in cognitive performance attributed to the volumetric changes in specific brain areas. All these changes rapidly aggravate under pathological conditions like Alzheimer’s disease (AD) (Harada et al., 2013). Previous findings from our lab showed that the age-related cognitive decline can be ameliorated by a 4-weeks treatment with the cannabinoid Δ^9^-tetrahydrocannabinol (THC). This cannabinoid is the major psychoactive ingredient in *Cannabis sativa*. It elicits its psychoactive effects by activating the cannabinoid receptor 1 (CB1), a main component of inhibitory synaptic feedback processes and synaptic plasticity, underlying learning and memory, reward, stress, and anxiety (Di Marzo et al., 2015; Lutz et al., 2015).

A role of CB1 signaling in the aging process has been suggested by genetic mouse models that show that the loss of CB1 receptors increased the mortality rate (Zimmer et al., 1999) and accelerated cognitive aging, accompanied by neuronal loss in the hippocampus and aging-like histological changes in the skin (Bilkei-Gorzo et al., 2005, 2012). The genetic data are probably physiologically relevant, as signaling properties of CB1 receptors and the endocannabinoid tone, declines during aging. Thus, CB1 receptor expression decreases in various brain regions and undergo functional alterations in ligand-binding and G protein-coupling with advancing age (Berrendero et al., 1998; Feliszek et al., 2016). Further studies from human postmortem brains reported a significant decrease in the expression of diacylglycerol lipase α (*Dagl*α), the synthesizing enzyme of 2-AG, after a young age (Long et al., 2012). Both 2-AG and DAGLα protein levels decrease prominently in the hippocampus of aged mice (Piyanova et al., 2015).

Enhancing CB1 receptor signaling in aged mice with a chronic low dose (3 mg/kg/day) THC improved the cognitive performance to levels reminiscent of young control mice. In contrast, the same dose in young mice resulted in cognitive impairment, similar to what was reported in human studies with young individuals. The beneficial cognitive effects of chronic THC are mediated through epigenetic modifications by enhancing histone acetylation in the hippocampus. THC-treatment also induced the transcription of *Bdnf* and *Klotho*, genes that improve synaptic plasticity and extend lifespan in aged mice, and strongly downregulated *Casp1* and connective tissue growth factor (*Ctgf*) genes, which regulate pro-aging processes (Bilkei-Gorzo et al., 2017). These animal findings suggest the intriguing possibility that cannabinoids could also ameliorate some forms of age- or disease-associated cognitive decline in humans.

In addition to medicinal marijuana, there are currently two forms of THC-based medications available: Dronabinol (Marinol, Syndros, Adversa, REDUVO), which consists primarily of THC and is available as capsules or in liquid formulations. Sativex^®^, administered as an oromucosal spray, is a 1:1 preparation of THC and cannabidiol. In our previous study, we have used a pure THC preparation. The goal of the present study is to compare the efficacy of THC with the concurrent administration of THC and CBD (1:1; THC:CBD), thus resembling Sativex^®^, on age-related cognitive decline in mice.

## Materials and Methods

### Animals

**FIGURE 1:**
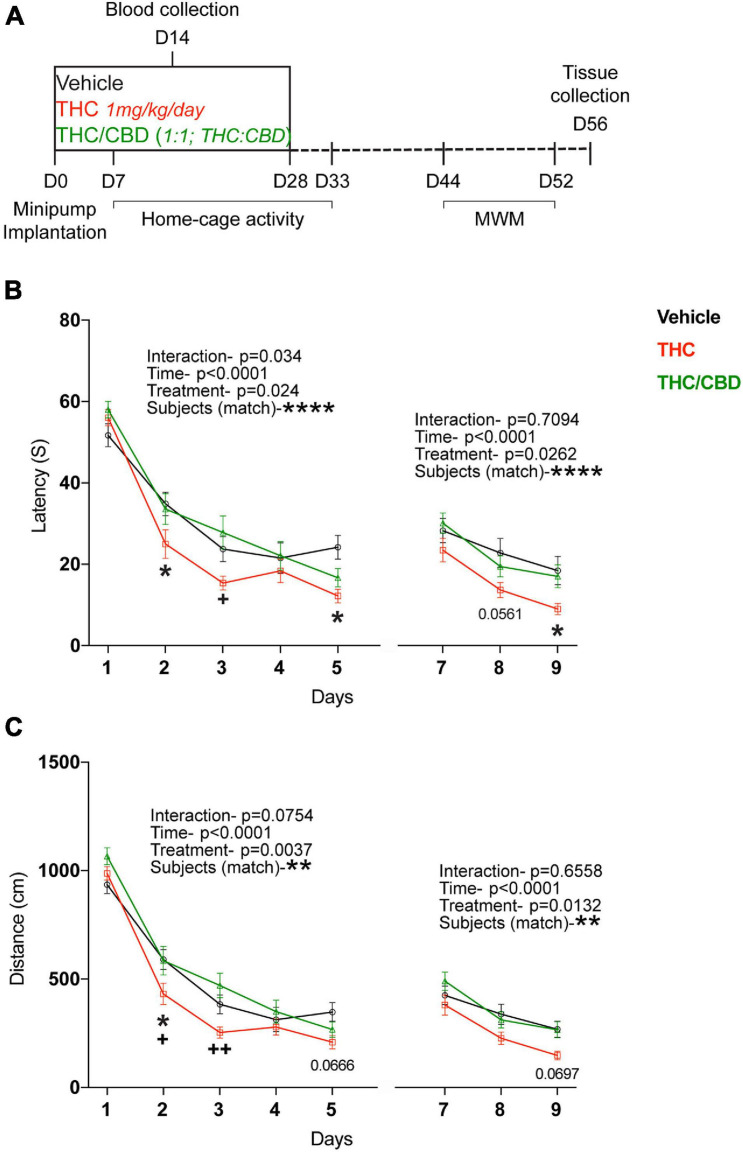
Effect of THC and THC/CBD on spatial learning in old mice. **(A)** Scheme depicting the experimental paradigm. **(B)** Spatial learning was assessed in the acquisition and reversal phases of the Morris water maze (MWM) test. Spatial learning performance was significantly improved in THC-group compared to vehicle- and THC/CBD-groups. **(C)** Spatial navigation ability tested during the acquisition and reversal phases of the MWM test. THC significantly improved the spatial navigation ability in old mice compared to vehicle- and THC/CBD-groups (*n* = 21–25 mice per group). Data represented as mean ± SEM, *p < 0.05 (difference between vehicle- and THC- treatment groups); ^+^*p* < 0.05, ^++^*p* < 0.005 (difference between THC-and THC/CBD-treatment groups); **p < 0.01; ****p < 0.0001 variation between the subjects; *p*-values stated are the differences between THC- and vehicle- treated groups; 2-way ANOVA (repeated measures) with Tukey’s multiple comparison test.

Old (12 and 18 month) male C57BL6/J mice were purchased from Janvier, France. The 18-month-old cohort (25 mice per group, altogether 75 mice) was used for the behavioral tests, whereas the 12-month-old cohort (8 per time point and treatment, altogether 48 mice) for the gene expression and metabolic studies. All animals were single caged and adapted to the reversed 12-h dark/light cycle for at least 2 weeks before the commencement of the experiments. Schedule of experiments is shown at Figure 1A. All animal procedures performed are in accordance with the European Communities Directive 86/609/EEC guidelines and are approved by the responsible state ministry, LANUV NRW, Germany (81-02.04.2018.A134).

### Pharmacological Treatments

We delivered either THC (1 mg/kg/day), or a 1:1 mixture of THC and CBD (THC/CBD, 1 mg/kg/day each) dissolved in a solvent (ethanol:cremophor:saline, 1:1:18, v/v/v), or the solvent alone (vehicle group) via Alzet^®^ osmotic minipumps (Charles River, Germany). The pumps were implanted subcutaneously into mice randomly assigned to the respective treatment groups. The osmotic minipumps released their content at a continuous rate for 28 days. The volume delivered was 2.64 μl/day. THC and CBD were obtained from THC-Pharm GmbH, Frankfurt am Main, Germany and GW Pharmaceuticals Ltd., Cambridge, United Kingdom.

### Surgical Implantation of Osmotic Minipumps

Mice were injected with 0.1 mg/kg buprenorphine subcutaneously at least 30 min prior to the surgery. Isoflurane was used to anesthetize the mice with an initial flow rate of 5% along with oxygen (5 L/min) and then maintained with 2% isoflurane for the duration of the procedure. The lateral region posterior to the scapulae was shaved, and a small incision was made on the skin. A pocket was created for the pump using a hemostat by spreading the subcutaneous tissue toward the rear side of the mouse but not to the extent that it impedes the hind limb movement. The pump was then inserted facing the flow moderator first to minimize contact between the drug delivered and the healing of the incision. The pump was gently pushed through the pocket, and the incision was then closed with the wound clips, leaving enough space to avoid tension or skin stretching around the wound. The animals were closely monitored until they completely revive from the anesthesia, and carprofen (4 mg/kg) was administered subcutaneously for the postoperative pain treatment.

### Home-Cage Activity

Post-operation, mice (18-month-old) were allowed to recover for a week and the home-cage activity was accessed (days 7–33). The home-cage activity of 9 mice per group was continuously monitored using a device with a built-in infrared motion sensor attached to the lid of cage top. The average activity of the mouse was recorded every 15 min by the Mouse-E-Motion—Universal data logger (Infra-e-motion GmbH, Germany). The cumulative activity monitored for each week was plotted separately and represented as the average activity per hour.

### Behavioral Analysis for Cognitive Performance

Old (18 month) mice were used to assess the spatial learning and memory in the Morris water maze (MWM) according to the protocols described earlier (Albayram et al., 2016; Bilkei-Gorzo et al., 2017). A circular pool (120 cm diameter and 60 cm depth) filled with opaque water (25°C) was separated into four equal quadrants. Pictures of various shapes and colors were fixed to the walls of the four quadrants thus acting as external cues and helping the mice to orient themselves. A transparent plexiglass platform was submerged in one of the quadrants for the mice to locate and escape. The task consists of acquisition (5 days), probe trial (1 day), and reversal phases (3 days). In the 5 days acquisition phase, mice were trained to locate the escape platform in the pool for four consecutive trials on each day with an inter-trial interval of 10 min. If the mice failed to locate the platform in 70 s they were gently guided to the submerged platform and let stand for 20 s. Mice were released from the same side of the pool on days 1 and 2 in all four trials, whereas on the remaining days they were released from different sides of the pool in each trial. During the probe trial (day 6), spatial memory was tested by removing the submerged platform from the pool and the time spent for each mouse searching the escape platform among the quadrants was assessed. In the reversal phase (last 3 days), the mice had to locate the submerged platform that was moved to the opposite quadrant of the pool. This test assesses the flexibility of learning. All experiments were captured using a roof fixed camera and the recordings were analyzed using an automatic tracking system (Ethovision XT, Noldus, Wageningen, Netherlands). Assessed were the escape latencies, an indicator of spatial learning and memory performance, as well as the length of the swim path, an indicator of spatial navigation. Animals showing wall hanging or circling (3-3 from the THC and THC/CBD groups) were excluded from the analysis.

### Sample Collection

Separate group of 12-month-old THC and THC/CBD treated mice were perfused to collect blood and tissue samples at various time points (days 28, 42, and 50). For this, mice were anesthetized with an intraperitoneal injection of a ketamine (100 mg/kg) and xylazine (20 mg/kg) mix and perfused with ice-cold PBS after ensuring the lack of any pain reflexes. Both hippocampus and visceral fat (lower abdominal region) were simultaneously isolated and flash-frozen in liquid nitrogen and stored at −80°C until further use. Blood was collected into EDTA tubes (inverted the tubes for mixing) right from the heart while the mice were under anesthesia and just before the perfusion. Whereas for the other cohort in which behavior was analyzed, blood samples were collected from the facial vein at day 14 (during the treatment) from the vehicle, THC, and THC/CBD treated mice. The blood samples were centrifuged at 2,000 × g for 15 min at 4°C, and the resultant plasma was carefully collected into fresh tubes, flash-frozen in liquid nitrogen, and stored at −80°C until further use. While both blood and visceral fat samples were used to determine the concentrations of THC, CBD, and THC metabolites, the hippocampal tissue was used for gene expression analysis.

### Gene Expression

Total RNA was extracted from the hippocampal tissue using TRIzol^TM^ reagent following the manufacturers protocol. After determining the RNA yield and quality, the samples were treated with RNase free DNase I (1 U/1 μg total RNA) to remove DNA contamination. Eventually the DNase I enzyme was inactivated by heating it to 75°C for 5 min. At least 1 μg RNA was reverse transcribed using SuperScript^TM^ II reverse transcriptase (Invitrogen^TM^) according to the manufacturers protocol and stored at −80°C for further use. TaqMan assays were used to determine the changes in the genes of interest, Mm01334042_m1 for *Bdnf*, Mm00438023_m1 for *Casp1*, and *Hprt* (Mm01545399_m1) was used as a reference gene. The changes in expression were analyzed using the competitive threshold cycle (ΔΔC_t_) method (Schmittgen and Livak, 2008).

### Determination of THC, 11-Hydroxy-Δ^9^-Tetrahydrocannabinol (11-OH-THC), 11-nor-9-Carboxy-Δ^9^-Tetrahydrocannabinol (THC-COOH) and CBD Levels

About 50 μl of plasma samples were spiked with 50 μl of methanolic internal standard mix [THC-D3 (200 ng/ml), 11-OH-THC-D3 (200 ng/ml), THC-COOH-D9 (200 ng/ml), and CBD-D3 (200 ng/ml)] (Cerilliant, Round Rock, TX, United States) and performed a liquid-liquid extraction by adding 500 μl of n-hexane/ethyl acetate (90:10, v/v) mixture. After a brief vortexing, samples were centrifuged (13,000 rpm, 10 min) and the organic phase was transferred to a separate tube. The sample residues were acidified with 50 μl of 0.1 M hydrochloric acid and re-extracted using 500 μl of n-hexane/ethyl acetate (90:10, v/v) mixture, and the organic phase was obtained as described above. Both the organic phases were combined and evaporated to dryness. They were reconstituted in a 50 μl mobile phase mixture (eluent A/eluent B, 10:90, v/v).

Approximately 30 mg frozen adipose tissue were added to Precellys^®^ tubes containing steel balls, 700 μl of an extraction mixture (n-hexane/ethyl acetate, 9:1, v/v), 50 μl of internal standard mix (as stated above), and 500 μl of 0.1 M formic acid. The contents were then homogenized using a Precellys^®^ 24 tissue homogenizer (one cycle, 20 s, 6,000 rpm). The resulting homogenates were subjected to centrifugation for phase separation. The upper organic phase was transferred to a separate tube and evaporated to dryness, and later resuspended in 100 μl of methanol and water (9:1, v/v) mixture. The samples were then vortexed for at least 10 min and centrifuged to collect the supernatants for the quantification of THC (along with its metabolites), and CBD.

### LC-QQQ-MS Analyses

THC, 11-OH-THC, THC-COOH, and CBD were quantified using high performance liquid chromatography (HPLC) coupled to triple quadrupole mass spectrometry (LC-QQQ-MS). The LC-QQQ-MS system consisted of a Shimadzu LC 20 series HPLC system (binary pump, degasser, column oven and autosampler) (Shimadzu, Duisburg, Germany) coupled to a Sciex API 4000 QTrap mass spectrometer (Sciex, Darmstadt, Germany).

Detection and quantification of cannabinoids was carried out using negative electrospray ionization and the multiple reaction monitoring mode. The following settings were used: collision gas: nitrogen, collision gas: high, curtain gas: 30 psi, ion source gas 1: 40 psi, ion source gas 2: 60 psi, ion spray voltage: -4500 V, temperature: 475°C. Used ion transitions and corresponding mass spectrometric potentials for the detection of analytes and internal standards are presented in Supplementary Table 1.

Chromatographic separation was achieved using a NUCLEODUR^®^ C18 Isis (5 μm, 4.6 × 150 mm) column from Macherey−Nagel (Dueren, Germany), hold at 40°C, and a 16-min run (total flow: 0.8 ml/min) using gradient elution. Eluents A and B were 5 mM ammonium acetate in deionized water (with 0.1 M acetic acid adjusted to pH 5.7) and acetonitrile/methanol (1:9, v/v, with 0.1 M acetic acid adjusted to pH 5.7). Injection volume was 30 μl.

Estimation of cannabinoid concentrations in plasma was achieved using matrix calibration (spiked blank plasma). Negative controls (blank plasma) as well as quality control samples (spiked blank plasma) were carried along each analysis sequence. Estimation of cannabinoid concentrations in adipose tissue was done preparing a solvent calibration. Negative controls (adipose tissue from untreated mice) as well as quality control samples prepared in solvent were carried along each analysis sequence.

### Data Analysis

The sample size in each group for behavioral assessment was determined using the power analysis before starting the experiment. Our analysis indicated an 85% probability of detecting an effect size of 33%. Statistical analysis was performed using GraphPad Prism 7 (STAT*CON* GmbH, Germany). Data depicting the changes with treatment and time as independent variables are analyzed using two-way analyses of variance (ANOVA) to determine differences between the groups. Statistical differences between two groups are analyzed using unpaired *t*-tests, and for the analysis of more than two groups we used one-way ANOVA. As a planned comparison, we compared treatment effects on the same day and effect of time within the same treatment groups using Tukey test for the water-maze and THC metabolism results. As a planned comparison for gene expression results Sidak’s test was used to compare the effect of time separately to the treatment groups. Data are represented as mean ± SEM, and the alpha level of statistical significance was set at *p* < 0.05. Significant outliers were verified and removed using the ROUT method. The group size for the individual data set is depicted in the corresponding figure legend.

## Results

### THC-Alone Is More Efficient in Improving Spatial Learning in Old Mice

Old (18-month-old) mice were chronically treated with either vehicle, THC (1 mg/kg/day) or THC/CBD (1 mg/kg/day THC and CBD each) using osmotic minipumps for 28 days. To determine if that dose influenced the activity of mice, we assessed the locomotor activity within the home-cage for a 3-week (days 7–33) period. As expected, there was a significant effect of time in the first week of testing (second treatment week) [*F*_(__165_,_3_,_630__)_ = 21.46; *p* < 0.0001], because the mice’s activity differs between the dark and the light phases. However, there was no significant effect of treatment [*F*_(__2_, _22__)_ = 0.3364; *p* = 0.7180] and no interaction [*F*_(__330__, 3_,_630__)_ = 0.8695; *p* = 0.9519]. The results in weeks 2 and 3 were similar (Supplementary Figure 1). This indicated that neither THC nor THC/CBD influenced the activity at the dose delivered.

After a washout period (2 weeks post-treatment cessation), the mice were tested for spatial learning in the MWM, by assessing the escape latencies. In the acquisition phase, all mice learned the location of the platform over time [main effect time, *F*_(__4_, _260__)_ = 97.9; *p* < 0.0001]. However, the acquisition rate was affected by the treatment [main effect treatment, *F*_(__2_, _65__)_ = 3.804; *p* = 0.0274; interaction *F*_(__8_, _260__)_ = 2.124; *p* = 0.0340]. Planned comparisons revealed a significant difference between the THC- and vehicle-treated groups on days 2 and 5, whereas there were no differences between the THC/CBD and vehicle groups. Further, THC-group performed significantly better on day 2 than the THC/CBD group. Together these data indicate an improved performance after THC treatment, but not after THC/CBD treatment.

The mice also learned the new location of the platform in the reversal phase [Time; *F*_(__2_, _138__)_ = 26.29; *p* < 0.0001]. Once again, we found a significant treatment effect [*F*_(__2_, _69__)_ = 3.842; *p* = 0.0262]. Planned comparisons showed a significant difference between the THC and vehicle groups on day 9 and a trend on day 8 (*p* = 0.0561), whereas there were no significant differences between the THC/CBD and vehicle groups (Figure 1B). This indicates that THC not only improved the spatial learning, but also the flexibility in learning.

The mice were assessed for the spatial memory during the probe-trial phase, on day 6, prior to the testing of flexibility in learning. All mice spent significantly longer time in the target quadrant indicating that all the mice have an intact spatial memory [quadrant, *F*_(__3_, _195__)_ = 41.15; *p* < 0.0001]. However, neither THC nor THC/CBD further improved spatial memory in old mice indicating no significant effects of treatment [*F*_(__2_, _65__)_ = 1.047; *p* = 0.3569] (Supplementary Figure 2).

We also analyzed the length of the swim path. In both the acquisition and reversal phases, over-time all mice navigated through significantly shorter swim paths to reach the hidden platform [main effect time, acquisition- *F*_(__4_, _260__)_ = 145.2; *p* < 0.0001; reversal- *F*_(__2_, _138__)_ = 27.49; *p* < 0.0001]. Again, we also observed a significant treatment effect in both phases of the test [acquisition- *F*_(__2_, _65__)_ = 6.104; *p* = 0.0037; reversal- *F*_(__2_, _69__)_ = 4.615; *p* = 0.0132]. Planned comparisons showed significant difference between the THC and vehicle groups only on day 2, whereas there were significant differences between THC and THC/CBD groups on days 2 and 3. We found no significant difference between the THC/CBD and vehicle groups (Figure 1C). Together these findings suggest spatial navigation was improved by the THC treatment, whereas concurrent administration of THC and CBD has no such an effect.

### Changes in THC Metabolism in the Presence of CBD

**FIGURE 2:**
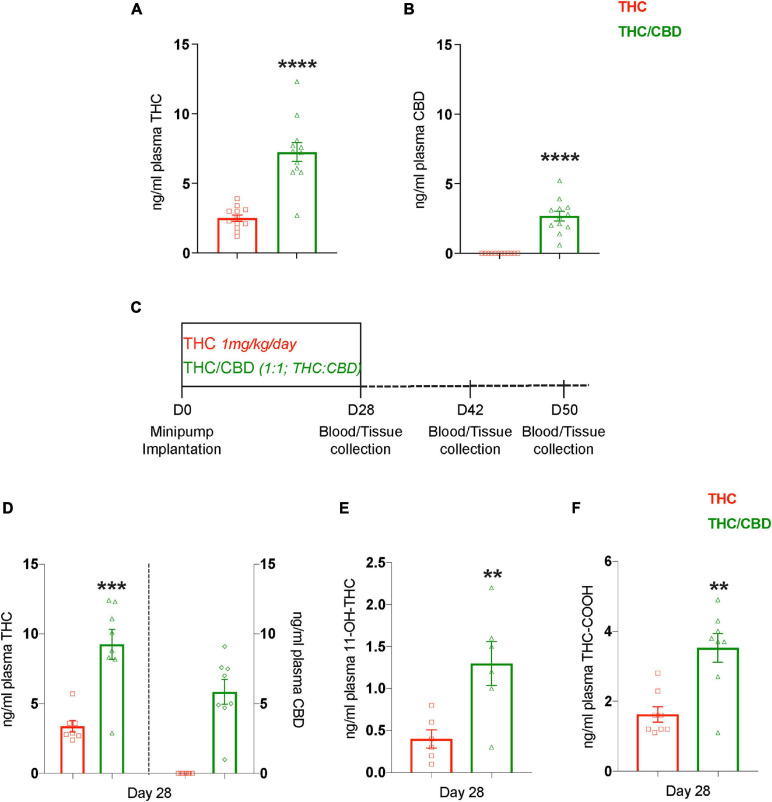
CBD elevates plasma THC levels. **(A,B)** Plasma concentrations of THC and CBD among the treatment groups measured 2 weeks (day 14) after the pump implantation shows that the presence of CBD elevates plasma THC levels (*n* = 12 mice per group). **(C)** The scheme depicts the time-course paradigm where mice were implanted with pumps on day 0, and tissue collected on day 28 (last day of treatment), day 42 (2 weeks post-treatment cease), and day 50 (3 weeks post-treatment cease). **(D)** Plasma THC and CBD concentrations measured on day 28 are depicted on left and right *Y*-axis respectively. Similar to day 14, the plasma THC levels are also elevated on day 28 in the presence of CBD (*n* = 7–8 mice per group). **(E,F)** Determining the concentrations of plasma THC metabolites showed an elevation in 11-OH-THC and THC-COOH in the presence of CBD. THC along with its metabolites, and CBD were not detected in the plasma at days 42 and 50 (*n* = 6–8 mice per group). Data represented as mean ± SEM, ****p < 0.0001; ***p < 0.0005; **p < 0.01; unpaired *t*-test.

We also measured the plasma levels of THC and CBD during the middle of the treatment (day 14) in the mice analyzed in the MWM. Interestingly, despite infusing the same dose of THC in both the treatment groups, the plasma THC levels in the THC/CBD group was approximately twice as high as in the THC group (*t*_22_ = 6.640; *p* < 0.0001) (Figure 2A). As expected, CBD was only detected in the THC/CBD group (Figure 2B). This difference indicated changes in THC metabolism in the presence of CBD. Therefore, we performed another experiment with an independent cohort of mice (12-month-old), to analyze plasma THC, CBD, and THC metabolites on the final day of the treatment (day 28), as well as later time points, days 42 (2 weeks post-treatment cease) and day 50 (3 weeks post-treatment cease). In addition, we also collected visceral fat tissue from the lower abdominal region and brain tissue (Figure 2C). In the plasma we found similarly increased THC levels (*t*_13_ = 4.78; *p* = 0.0004) in the THC/CBD group compared to the THC group, as observed previously at day 14 (left *Y*-axis; Figure 2D). Further, CBD was only detected in the THC/CBD group (right *Y*-axis; Figure 2D). In addition, 11-OH-THC (*t*_10_ = 3.195; *p* = 0.0096) and THC-COOH (*t*_14_ = 4.093; *p* = 0.0011), the primary and the most abundant metabolites of THC, were also significantly elevated in the THC/CBD group in comparison to the THC group (Figures 2E,F). These results show that the CBD effects on THC levels were sustained until the last day of the treatment and indicate that the presence of CBD alters the pharmacokinetics of THC. At later time-points, days 42 and 50 neither THC nor its metabolites nor CBD were detected in the plasma samples.

**FIGURE 3:**
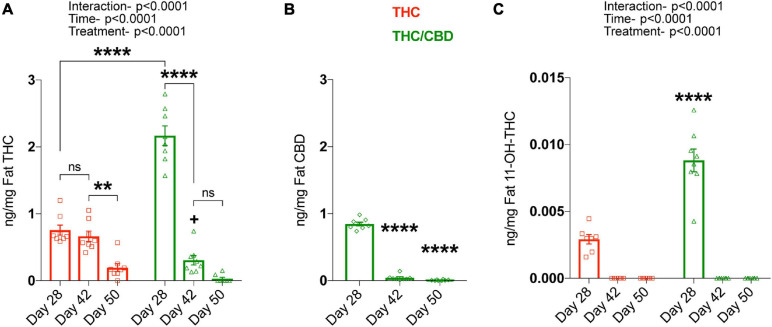
CBD inhibits THC metabolism. Fat THC and its metabolites as well as CBD concentrations were determined at various time-points, on the last treatment day (day 28), 2 weeks post-treatment (day 42), and 3 weeks post-treatment (day 50). **(A)** Fat THC levels were significantly altered based on time and the type of treatment. Data represented as mean ± SEM, **p < 0.01; ****p < 0.0001; ^+^*p* < 0.05 (difference in fat THC levels at day 42 between THC and THC/CBD groups); 2-way ANOVA with Tukey’s multiple comparison test. **(B)** Fat CBD concentrations in the THC/CBD group significantly decreased over-time. Data represented as mean ± SEM, ****p < 0.0001 (difference in fat CBD levels between day 28 and day 42 or day 50); 1-way ANOVA with Tukey’s multiple comparison test. **(C)** Increased fat 11-OH-THC, metabolite of THC in the presence of CBD at day 28. No fat THC metabolites are detected at day 42 and day 50 (*n* = 7–8 mice per group). Data represented as mean ± SEM, ****p < 0.0001 (difference in fat 11-OH-THC levels at day 28 between THC and THC/CBD groups); 2-way ANOVA with Tukey’s multiple comparison test.

As THC, CBD, and THC metabolites accumulate in fat tissues after chronic treatment, we next analyzed these tissues. For THC, we found significant main effects for time [*F*_(__2_, _42__)_ = 140.2; *p* < 0.0001) and treatment [*F*_(__1_, _42__)_ = 19; *p* < 0.0001], as well as an interaction [*F*_(__2_, _42__)_ = 67.96; *p* < 0.0001], as the amount of THC in both treatment groups altered temporally, and in a treatment specific-manner. Planned comparisons showed significantly higher THC levels in the THC/CBD group compared to the THC group at day 28, which resembles the plasma THC levels. The clearance of THC from the fat was relatively slow in the THC group, showing no significant changes between days 28 and 42. On day 50, a small amount of THC was clearly detectable. In contrast, THC was more rapidly cleared from the fat in the THC/CBD group. Thus, we found that on day 42 the THC level was already significantly reduced and on day 50 THC was barely detectable. The more rapid clearance of THC in the THC/CBD group is also indicated by the fact that THC levels were significantly lower than in the THC group on day 42, which is opposite to the difference on day 28 (Figure 3A). CBD in THC/CBD group was also rapidly cleared after day 28 and barely detectable at day 50 [*F*_(__2_, _21__)_ = 711.5; *p* < 0.0001] (Figure 3B).

Determination of 11-OH-THC levels in the fat of THC and THC/CBD groups across the time-points showed a significant effect of time [*F*_(__2_, _41__)_ = 156.7; *p* < 0.0001], treatment [*F*_(__1_, _41__)_ = 40.54; *p* < 0.0001], and interaction [*F*_(__2_, _41__)_ = 39.62; *p* < 0.0001]. The planned comparisons showed that the presence of CBD significantly elevated fat 11-OH-THC at day 28 in contrast to THC group. No fat 11-OH-THC was detected at later time-points (Figure 3C). Together these findings indicate that the presence of CBD alters the dynamics of THC metabolism.

### Time-Course of THC- and THC/CBD-Induced Gene Expression

**FIGURE 4:**
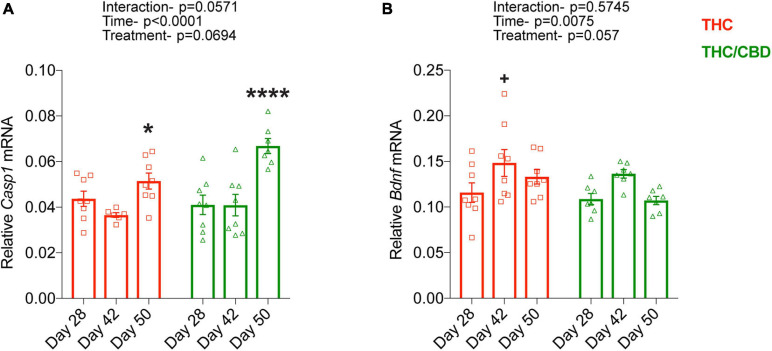
Time-course of THC- and THC/CBD-induced gene expression. **(A,B)** Time dependent changes in *Casp1* and *Bdnf* expression in THC- and THC/CBD groups in the hippocampus of old mice. A significant time-dependent but not treatment specific changes in the expression of *Casp1* and *Bdnf* were observed in both THC- and THC/CBD-groups. **(A)** *Casp1* expression moderately and substantially increased in THC-and THC/CBD-groups, respectively at day 50. **(B)** Whereas, *Bdnf* expression significantly increased at day 42 in THC-group but not in THC/CBD-group (*n* = 6–8 mice per group). Data represented as meam ± SEM, *p < 0.05 (difference between days 42 and 50 of THC-group), ****p < 0.0001 (difference between day 28/day 42, and day 50 of THC/CBD-group), ^+^*p* < 0.05 (difference between days 28 and 42 of THC-group); 2-way ANOVA with Sidak’s multiple comparison test.

Finally, we determined the time course of THC- and THC/CBD-induced changes in the expression of two genes, *Casp1* and *Bdnf*, which we previously found to be oppositely regulated by chronic THC exposure in the hippocampus of old mice. THC and THC/CBD treatment similarly influenced the expression of *Casp1* [treatment effect: *F*_(__1_, _39__)_ = 3.486; *p* = 0.0694]. *Casp1* expression level was enhanced after the termination of the drug treatments [time effect: *F*_(__2_, _39__)_ = 16.88; *p* < 0.0001] in both treatment groups [treatment × genotype interaction: *F*_(__2_, _39__)_ = 3.084; *p* = 0.0571]. Planned comparisons revealed a moderate increase which reached the level of significance in the THC group, while there was a substantial increase in THC/CBD group at day 50 (Figure 4A). *Bdnf* expression was again similarly altered by THC and THC/CBD groups [treatment effect: *F*_(__1_, _39__)_ = 3.847; *p* = 0.0570]. Its expression was elevated at day 42, in the early withdrawal phase [time effect: *F*_(__2_, _39__)_ = 5.556; *p* = 0.0075]. Although the lack of significant interaction between treatment and time [*F*_(__2_, _39__)_ = 0.562; *p* = 0.574] suggests that this effect was similar between the treatment groups, planned comparisons revealed that this increase was significant in THC-treated but not in the THC/CBD-treated animals (Figure 4B).

## Discussion

In the present study we determined the efficacy of THC alone as well as in combination with CBD in alleviating the cognitive decline in aged mice. Our findings show that THC alone, surprisingly, is more efficacious than the concurrent administration of THC and CBD in 1:1 ratio, same as in Sativex^®^. Thus, a dose of 1 mg/kg/day THC improved spatial learning in old mice in the MWM, whereas the same dose of THC co-administered with 1 mg/kg/day of CBD had no effects.

The results were surprising to us, because previous studies administering THC alone or in combination with CBD in a transgenic AD mouse model showed that THC/CBD was similarly effective in improving cognitive performance as THC alone in an object recognition test. THC/CBD was even more effective in reducing the production of soluble Aβ42 in an Alzheimer mouse model than THC alone (Aso et al., 2015, 2016). Further, it has been shown that chronic THC administration (3 mg/kg/day) to young mice impaired cognitive performance in a novel object recognition paradigm and that this detrimental effect was prevented by co-administration of CBD (Murphy et al., 2017). Furthermore, both short and long-term use of THC/CBD, but not THC alone, was shown to be efficacious in alleviating the intractable pain in patients with advanced stages of cancer (Johnson et al., 2010, 2013). Interestingly, the metabolic interaction of THC and CBD, when administered simultaneously, seems to influence the beneficial effects of THC. We here show that the presence of CBD at a similar dose to that of THC altered the pharmacokinetics of THC in the THC/CBD group ensuing in elevated THC levels during treatment, but a more rapid clearance of THC post-treatment.

Potentiating CB1 receptor signaling using a chronic low dose (3 mg/kg/day) of THC, a dose where none of the psychoactive side effects were detected, alleviated the cognitive decline associated with physiological aging in mice (Bilkei-Gorzo et al., 2017). Indeed, the THC dose (3 mg/kg/day) used earlier was quite efficient and improved the cognitive performance of aged mice to a level on par with the control young mice. However, we chose to use a much lower THC dose (1 mg/kg/day) and the combination of THC and CBD (each 1 mg/kg/day) in the present study because we expected a higher efficacy of the combination and wanted to avoid a ceiling effect. Further, we also wanted to minimize the influence of a higher dose of THC and CBD combination on their metabolizing enzymes, cytochrome P450’s (CYPs). We adopted the same treatment paradigm that elicited the long-lasting beneficial effects of THC (Bilkei-Gorzo et al., 2017), apart from the dose and the longer washout periods prior to behavior analysis.

It is important to note that the beneficial effects of THC on cognitive performance are extremely age-dependent. However, it is also beneficial under pathological conditions like AD, even at a young age. As described earlier, a chronic low dose THC (3 mg/kg/day) restored the learning and memory performance of old mice as tested in a battery of behavioral tests (MWM, novel object location recognition, and social recognition tests) (Bilkei-Gorzo et al., 2017). Consistent with the above findings, we found that prolonged exposure to THC prominently improved the learning abilities in aged mice despite using a lower THC dose (1 mg/kg/day). Similarly, findings showed that THC-treatment restored memory deficits in transgenic mice with AD-like pathology even at an early symptomatic stage (Aso et al., 2015). Interestingly, although repeated CBD-treatment *per se* has no effect on cognitive performance in young mice, pre- or co-treatment of CBD was shown to antagonize the THC-induced behavioral abnormalities in young humans and mice (Bhattacharyya et al., 2010; Murphy et al., 2017; Schleicher et al., 2019). Further, CBD-treatment alone significantly restored the memory in mice with AD-like pathology at an early symptomatic stage (Aso et al., 2015). However, CBD’s ability to improve the age-dependent cognitive decline is still an open question and needs further investigation. Evidence suggests that chronic exposure to the combination of THC and CBD efficiently reduced the learning impairment and also resulted in a marked reduction in gliosis than THC or CBD separately in mice with AD-like pathology (Aso et al., 2015). Later, the same group also demonstrated that the combination of THC and CBD was also efficient in reducing the memory impairment in aged mice with AD-like pathology at advanced stages of the disease. Conversely, the same study reported that THC and CBD combination could not restore the memory impairment caused by physiological aging in mice (Aso et al., 2016). In accordance with this, our data also suggest that the concurrent administration of THC and CBD (each 1 mg/kg/day) is less effective in improving the learning ability in aged mice than THC alone.

The ability of mice to precisely locate and navigate to the hidden platform in the MWM test requires proper topographical orientation using the distal cues. This process mainly involves the neuronal circuitry of the hippocampus and the entorhinal cortex and is vulnerable to age-related changes. Indeed, alterations in navigational abilities may even serve as a sensitive marker of impending cognitive decline associated with neuropathological conditions or advancing age (Moffat et al., 2001; Vorhees and Williams, 2014; Lester et al., 2017). A recent study stated an impairment in spatial navigational abilities as early as 2 months of age in a transgenic AD mouse model (Karunakaran, 2020). In our study, we show that prolonged exposure to THC but not THC/CBD in old mice significantly improved the spatial navigation.

Surprisingly, despite possessing two times more THC in the blood of THC/CBD group during the treatment phase, concurrent administration of THC and CBD failed to improve the spatial learning abilities in aged mice compared to vehicle group. There are three possible mechanisms that might explain the above behavioral findings: (a) CBD as an antagonist at G protein-coupled receptor 55 (GPR55) in the brain, (b) the fact that CBD acts as a negative allosteric modulator at the CB1 receptor, and (c) the metabolic interaction between THC and CBD in the liver and fat. In the following, we are discussing these possibilities.

GPR55, a cannabinoid-like receptor, with lysophosphatidylinositol (LPI) being its endogenous ligand is one of the pharmacological targets of CBD. Evidence suggests that GPR55 regulates cognitive processes independent of CB1 signaling. Blockade of GPR55 in the dorsolateral striatum was shown to impair procedural memory (Marichal-Cancino et al., 2016), whereas pharmacological stimulation of hippocampal GPR55 was shown to regulate spatial learning and memory processes (Marichal-Cancino et al., 2018). Further, LPI-mediated activation of GPR55 was shown to enhance the synaptic release probability in the CA3-CA1 synapses, the effect that is blocked by CBD (Sylantyev et al., 2013). The antagonistic effect of CBD at GPR55 may have contributed to the lack of improvement in spatial learning in aged mice treated with THC/CBD.

Recent studies claim that CBD acts as a negative allosteric modulator at the CB1 receptor, through which it could possibly antagonize the THC effects. The binding of CBD to the allosteric site of the CB1 receptor is shown to inhibit two forms of synaptic plasticity, depolarization-induced suppression of excitation, and metabotropic suppression of excitation (Laprairie et al., 2015; Straiker et al., 2018). However, our findings from the time-course of THC- and THC/CBD-induced gene expression are in contrast to this notion. The cognitive decline in the aging process is regulated by the increased expression of caspase-1, a protein that regulates the maturation and secretion of interleukin (IL)-1β and IL-18, and decreased expression of BDNF, a protein that improves synaptic connectivity (Gemma and Bickford, 2007; Erickson et al., 2011; Neidl et al., 2016; Mejias et al., 2018). THC-treatment was shown to reverse the molecular clock by strongly downregulating the expression of *Casp1* and inducing the expression of *Bdnf* in aged mice (Bilkei-Gorzo et al., 2017). Confirming the above findings, we saw a significant time-dependent changes in the expression of *Casp1* and *Bdnf* with THC (1 mg/kg/day) in aged mice. Interestingly, THC/CBD was also able to modulate the expression of *Casp1* and *Bdnf* in a temporal fashion suggesting that CBD presence has considerably less impact on THC-induced gene expression.

So far, several studies using different routes of administration have shown that either pretreatment or co-administration of CBD increased the bioavailability of THC, illustrated by elevated blood THC levels (Bornheim et al., 1995; Huestis, 2007; Britch et al., 2017; Hložek et al., 2017; Murphy et al., 2017). In accordance with these findings, we saw a significant elevation of plasma THC levels during the treatment phase in the THC/CBD group compared to THC-alone group. It is likely that the elevated plasma THC levels are due to a modulation of THC metabolism by CBD.

The plasma levels of the THC and CBD are mostly influenced by the activity of CYP enzymes. In humans, the enzymes that majorly catalyze the oxidative metabolism of THC and CBD are CYP2C9, CYP2C19, and CYP3A4 (Zendulka et al., 2016). THC is consecutively hydroxylated to form the primary and most abundant metabolites, 11-OH-THC (psychoactive) and THC-COOH (psycho-inactive), respectively in the first phase of metabolism. In the second phase THC-COOH forms conjugate with glucuronic acid, which increases its aqueous solubility and facilitates the excretion (Sharma et al., 2012). Interestingly, THC, and to a greater extent, CBD have an inhibitory effect on CYP activity. Further, the combination of 1:1, THC:CBD induces a substantially higher inhibitory effect on CYP activity than THC- and CBD- independently (Barnes, 2006). During the treatment phase, plasma samples from THC/CBD-treated mice showed a significant elevation of THC and its metabolites, 11-OH-THC and THC-COOH compared to THC alone treated mice. This observation is consistent with the reported inhibitory effect of THC/CBD on CYPs activity. Nevertheless, THC and its metabolites were eventually cleared from the plasma, as neither THC nor its metabolites could be detected at day 42 (2 weeks post-treatment) and day 50 (3 weeks post-treatment). Clearance of the highly lipophilic THC and CBD from the plasma are not only achieved by metabolism, but also through absorption and storage in fat tissues. Both THC and CBD can be detected for an extended period of time in these tissues, because they are only released into the blood stream and metabolized in the liver at a low rate. This process can be enhanced through lipolysis induced by physical exercise, food deprivation, and elevated adrenocorticotropic hormone activity (Gunasekaran et al., 2009; Wong et al., 2013; Foster et al., 2019). Consistent with our analysis of THC in the plasma, we also found elevated THC levels in fat tissues of the THC/CBD group at day 28. Interestingly, however, the clearance rate of THC from fat tissue (ratio of THC levels on days 28 and 42) was higher in the THC/CBD group, compared to the THC-alone group. This effect cannot be explained by a CYP inhibition of THC/CBD combination alone, but also suggests that CBD may modulate the clearance of THC from fat stores, via an unknown mechanism. The compensatory mechanisms due to long-term inhibition of CYPs activity resulting in increased expression and activity of CYPs could be another possible explanation for the rapid clearance of long-accumulated THC in the THC/CBD group. Oxidation of THC and its metabolites decreases their lipophilicity and affects their fat reabsorption. Therefore, the only metabolite detected in the fat was 11-OH-THC and not THC-COOH. Similar to our earlier observations in plasma, the fat 11-OH-THC levels were also elevated in the THC/CBD group at day 28. The lipophilic nature of CBD is moderately less compared to THC (Morales et al., 2017). This could affect the fat absorption or storage of CBD and may have contributed to the rapid clearance of CBD from the fat tissue before day 42.

We cannot entirely exclude the possibility that the diminished efficacy of drug treatment in the THC/CBD group was due to the increased THC plasma levels. However, in our previous study, we found that a three times higher dose of 3 mg/kg/day THC dose was very effective in reversing the cognitive decline in both 12 and 18 month old mice (Bilkei-Gorzo et al., 2017). The elevated plasma THC levels in the THC/CBD group (in the current study) very likely well correspond to the plasma THC levels of 3 mg/kg/day THC treated mice, the dose that still reversed the age-dependent cognitive decline.

In conclusion, our observations indicate that 1 mg/kg/day THC dose is still effective in improving the spatial learning in aged mice. With regard to the efficacy, THC-alone has proved to be more efficient in improving spatial learning in aged mice than its 1:1 combination with CBD. However, the possibility of THC/CBD being efficient in other ratios or at the earliest time-points, like immediately after the treatment cease, cannot be negated. Possibly, reducing the dose of CBD may improve the efficacy of the THC/CBD combination. The lack of improvement in spatial learning of THC/CBD group could be due to the time-point at which behavior was analyzed. At this time-point (day 44 = more than 2 weeks post-treatment cease), the amount of THC present in the THC/CBD group was substantially low compared to the THC alone group. Further, the relatively transient effects of THC/CBD can be attributed to CBD, as the presence of CBD altered the pharmacokinetics of THC, causing the beneficial effects of THC/CBD to wane off more swiftly compared to the longer-lasting effects of THC-alone.

## Data Availability Statement

The raw data supporting the conclusions of this article will be made available by the authors, without undue reservation.

## Ethics Statement

The animal study was reviewed and approved by the Landesamt für Natur, Umwelt und Verbraucherschutz Nordrhein-Westfalen (LANUV), NRW, Germany (approval number: 81-02.04.2018.A134).

## Author Contributions

PN, AB-G, and AZ contributed to conception and design of the study, interpreted the results, and drafted the manuscript. PN, AB-G, MK, BS, MP, and EB performed the experiments and analyzed the data. MK wrote sections of the manuscript. BM partly provided the resources. AB-G and AZ acquired the funding. All authors critically reviewed and approved the submitted version of the manuscript.

## Conflict of Interest

The authors declare that the research was conducted in the absence of any commercial or financial relationships that could be construed as a potential conflict of interest.

## Publisher’s Note

All claims expressed in this article are solely those of the authors and do not necessarily represent those of their affiliated organizations, or those of the publisher, the editors and the reviewers. Any product that may be evaluated in this article, or claim that may be made by its manufacturer, is not guaranteed or endorsed by the publisher.

## Abbreviations

AD: Alzheimer’s disease
2-AG: 2-arachidonoylglycerol
BDNF: Brain-derived neurotrophic factor
CB1: Cannabinoid receptor 1
CBD: Cannabidiol
CTGF: Connective tissue growth factor
CYP: Cytochrome P450
Daglα: Diacylglycerol lipase α
ECS: Endocannabinoid system
IL: Interleukin
MWM: Morris water maze
THC: Δ ^9^-tetrahydrocannabinol
11-OH-THC: 11-hydroxy-Δ^9^-tetrahydrocannabinol
THC-COOH: 11-nor-9-carboxy-Δ^9^-tetrahydrocannabinol.
