# Supplementary figures and images for: Efficacy of Δ9 -Tetrahydrocannabinol (THC) Alone or in Combination With a 1:1 Ratio of Cannabidiol (CBD) in Reversing the Spatial Learning Deficits in Old Mice
Source: Front Aging Neurosci. 2021 Aug 30;13:718850. doi: 10.3389/fnagi.2021.718850 (PMC8435893; doi:10.3389/fnagi.2021.718850)

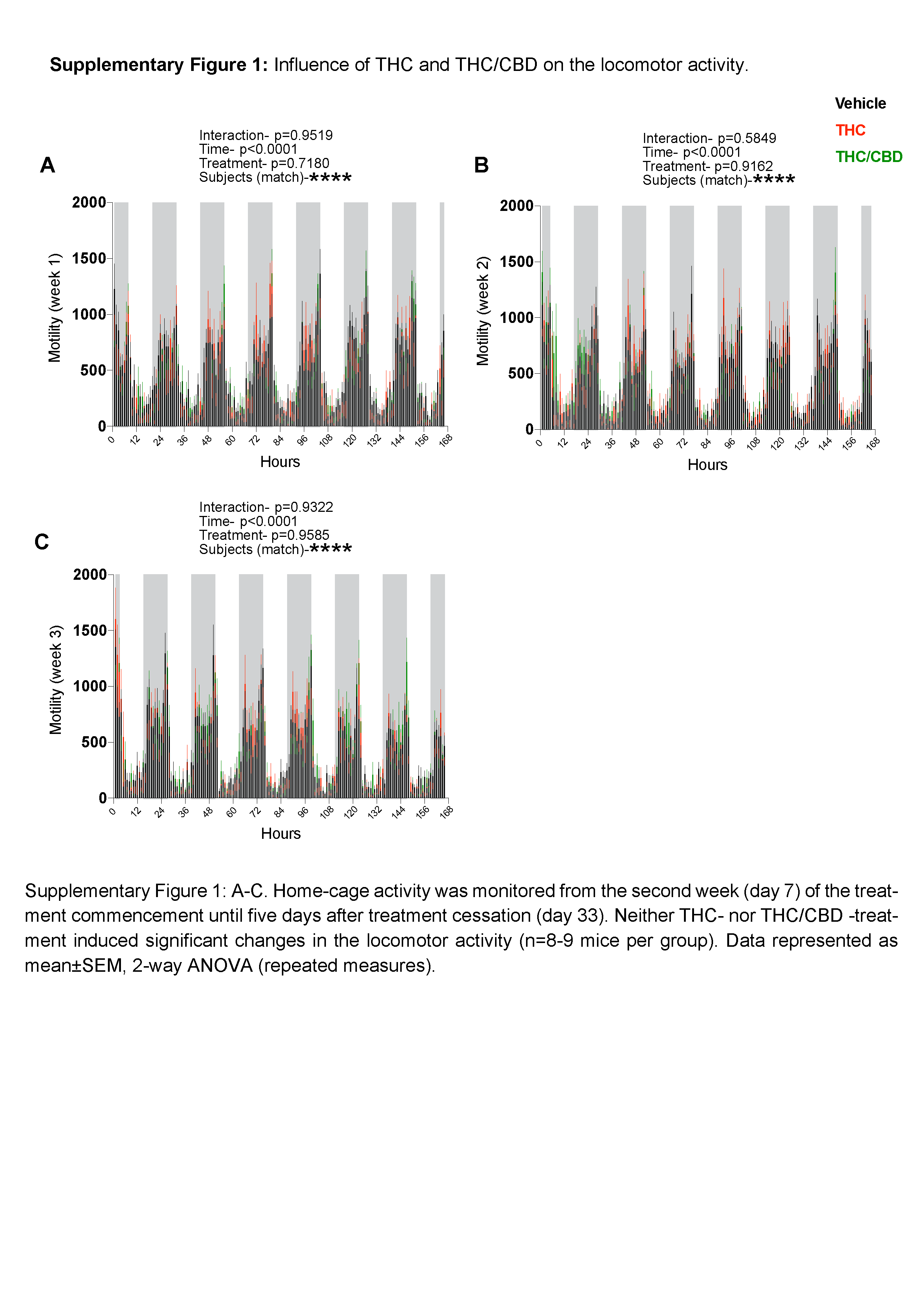

Supplement: Supplementary file 1 [file Image_1.TIF]

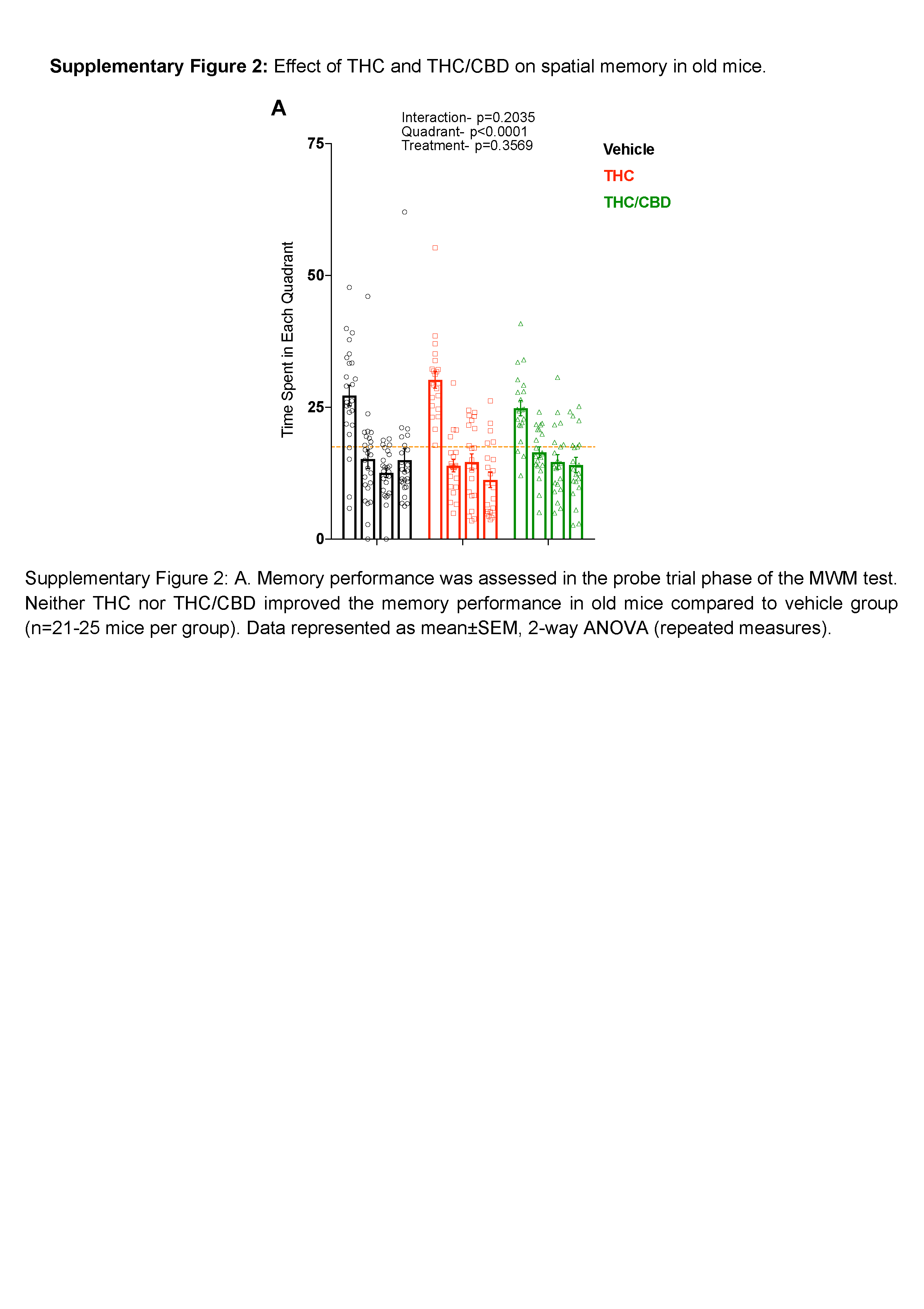

Supplement: Supplementary file 2 [file Image_2.TIF]
