# Supplementary material for: Efficacy of Δ9 -Tetrahydrocannabinol (THC) Alone or in Combination With a 1:1 Ratio of Cannabidiol (CBD) in Reversing the Spatial Learning Deficits in Old Mice
Source: Front Aging Neurosci. 2021 Aug 30;13:718850. doi: 10.3389/fnagi.2021.718850 (PMC8435893; doi:10.3389/fnagi.2021.718850)
Supplement: Supplementary file 3 [file Table_1.docx]

**Supplementary Table-1:** Ion transitions and corresponding mass spectrometric potentials.

| **Component** | **ion transition [m/z]** | **declustering potential [V]** | **entrance potential [V]** | **collision energy [V]** | **collision cell exit potential [V]** |
| --- | --- | --- | --- | --- | --- |
| CBD (target) | 313.3 / 245.2 | -97.09 | -10.00 | -30.90 | -3.75 |
| CBD (qualifier) | 313.3 / 179.2 | -97.09 | -10.00 | -28.98 | -6.94 |
| CBD-D3 (target) | 316.2 / 182.1 | -99.39 | -10.00 | -28.94 | -2.35 |
| CBD-D3 (qualifier) | 316.2 / 248.1 | -99.39 | -10.00 | -29.06 | -4.22 |
| delta9-THC (target) | 313.2 / 245.0 | -111.07 | -10.00 | -39.01 | -1.32 |
| delta9-THC (qualifier) | 313.2 / 191.1 | -111.07 | -10.00 | -38.00 | -7.16 |
| THC-D3 (target) | 316.2 / 248.1 | -115.00 | -10.00 | -36.96 | -4.35 |
| THC-D3 (qualifier) | 316.2 / 194.1 | -115.00 | -10.00 | -35.94 | -3.35 |
| 11-OH-THC (target) | 329.1 / 310.9 | -73.98 | -10.00 | -30.14 | -28.82 |
| 11-OH-THC (qualifier) | 329.0 / 172.9 | -73.98 | -10.00 | -46.93 | -2.88 |
| 11-OH-THC-D3 (target) | 332.2 / 314.2 | -95.68 | -10.00 | -28.75 | -5.97 |
| 11-OH-THC-D3 (qualifier) | 332.2 / 271.3 | -85.02 | -10.00 | -40.11 | -17.14 |
| THC-COOH (target) | 343.1 / 245.2 | -84.97 | -10.00 | -39.29 | -10.07 |
| THC-COOH (qualifier) | 343.1 / 191.3 | -84.97 | -10.00 | -45.98 | -11.79 |
| THC-COOH-D9 (target) | 352.2 / 254.2 | -103.78 | -10.00 | -39.25 | -14.79 |
| THC-COOH-D9 (qualifier) | 352.2 / 308.2 | -103.78 | -10.00 | -30.56 | -17.80 |
